# Microbial decay of wooden structures: actors, activities and means of protection

**DOI:** 10.1007/s00253-025-13443-z

**Published:** 2025-03-05

**Authors:** Lauritz Schrader, Christian Brischke, Jochen Trautner, Christoph C. Tebbe

**Affiliations:** 1 Thünen Institut Für Holzforschung, Leuschnerstraße 91, 21031 Hamburg, Germany; 2 Thünen Institut Für Biodiversität, Bundesallee 65, 38116 Braunschweig, Germany

**Keywords:** Biodegradation, Timber wood, Lignocellulose, Decomposition, Wood DNA, Microbial diversity

## Abstract

**Abstract:**

Wood decay fungi and bacteria play a crucial role in natural ecosystems, contributing to the decomposition of lignocellulosic materials and nutrient cycling. However, their activity poses significant challenges in timber durability, impacting industries reliant on wood as a construction material. This review examines the diversity of microorganisms damaging timber used indoors and outdoors. Additionally, traditional and advanced methods for microbial identification are discussed, with a focus on DNA-based, culture-independent sequencing methods whose importance has increased massively in recent years. It also provides an overview of the various options for wood protection, starting from wood protection by design, to chemical wood preservation and wood modification methods. This should illustrate how important it is to combine an ecological understanding of the decay organisms, precise identification and innovative wood protection methods in order to achieve a long-term and thus resource-saving use of wood.

**Key points:**

• *Fungi and bacteria play a crucial role in the decomposition of timber wood.*

• *Traditional and advanced DNA-based methods for microbial identification are discussed.*

• *An overview of the various options for wood protection is provided.*

## Introduction

In recent years, the increased use of construction timber has been promoted in Europe by several national initiatives (e.g. Austrian Federal Ministry of Agriculture, Forestry, Regions and Water Management 2022; Finish Ministry of Environment 2016; German Federal Ministry for Housing, Urban Development and Building and German Federal Ministry of Food and Agriculture 2023). Due to its low environmental impact and reduced greenhouse gas emissions compared to other building materials such as cement or steel, the use of timber is seen as one way of achieving the goals set by the Paris Agreement or the European Green Deal (Di Ruocco and Gaita 2023; Dodoo et al. 2014; European Commission 2019; Hafner and Schäfer 2017; United Nations 2015a). However, the susceptibility of wood to wood-decaying microorganisms poses a challenge for its sustainable use. For characterizing the hazards and risks of microbial decomposition of wood material, the contrasting conditions between indoor and outdoor exposure must be considered. Wood used indoors is usually permanently dry and protected from the varying weather conditions, which inhibits microbial degradation, since the micro-organisms require wood moisture content to be above plant cell wall saturation for their degradation mechanisms to work. Due to the higher humidity and exposure to precipitation timber used outdoors is much more at risk of premature failure (Humar and Thaler 2017; Ribera et al. 2017; Martín and López 2023).

In this review, we provide a combined overview of identification methods to characterize and monitor wood decay organisms as well as different possibilities of wood protection and wood modification with the objective of reducing the susceptibility of wood material to decaying microorganisms and thereby enhancing its service life.

## Wood decaying microorganisms

### Fungi

Fungi, especially filamentous basidiomycetes and, to a lesser extent, also ascomycetes, are mainly responsible for the degradation of wood (Baldrian and Valásková 2008; Hammel 1997). While their ability to degrade wood is of great ecological importance in the carbon cycle and functionality of terrestrial ecosystems (Watkinson et al. 2006), fungi cause enormous economic damage of the built environment. In France, restoration costs for damage to houses caused by wood-destroying fungi have been estimated at more than 30 million € per year (Maurice et al. 2011). In Germany and Switzerland, annual costs of 36 million € were reported for the replacement of prematurely failing utility poles (Ribera et al. 2017) and in the US annual timber damage by fungi is estimated at 1 billion dollar (Kirker 2018).

Wood-destroying fungi are categorized, irrespective of their phylogeny, based on their mode of degradation of the cell wall of the wood material and the resulting macroscopic and microscopic decomposition patterns as white, brown and soft rot fungi (reviewed in Blanchette 2000; Daniel 2016; Schmidt 2006). Briefly, white rot fungi degrade the plant cell wall from the lumen outwards by releasing enzymes from hyphae which attack all main components (cellulose, hemicellulose and lignin; Schmidt 2006). For the degradation of cellulose enzymes like endoglucanases, cellobiodydrolases and β-glucosidases are produced; the decomposition of hemicelluloses requires a wide set of enzymes (e.g. endoxylanase, endomannanase, β-mannosidase) due to their structural diversity and lignolytic systems consist of oxidases, peroxidases and hydrogen peroxide producing enzymes (Baldrian 2008). White rot is divided into two types based on the sequence of degradation of the main components (i.e. cellulose, hemicellulose and lignin). In the case of simultaneous white rot, all three components are degraded nearly at the same rate (Schwarze 2007). Whereas in the case of selective white rot, lignin is degraded earlier in the decay process than cellulose and hemicellulose (Schwarze 2007). In contrast, brown rot fungi do not produce lignolytic enzymes and therefore deploy a combination of oxidative and enzymatic degradation. First, non-enzymatic compounds like oxalic acid diffuse into the plant cell wall, disrupt the lignocellulose complex by generating hydroxyl radicals and facilitate the penetration of hydrolytic enzymes (e.g. endoglucanases, β-glucosidases) to break down cellulose and hemicelluloses (Arantes and Goodell 2014; Arantes et al. 2012; Baldrian and Valásková 2008). Soft rot fungi penetrate the S_2_-layer of the cell wall with fine hyphae, which branch out parallel in the orientation of cellulose microfibrils and secrete cellulose-degrading enzymes (e.g. cellulases, endoglucanases, β-glucosidases) producing typical cavities as a result (Schmidt 2006; Schwarze 2007; Simonis et al. 2008). This cavity-forming degradation pattern is also known as Type I soft rot, and if, in addition to the cavities, the cell wall is eroded by hyphae growing in the lumina, it is known as Type II soft rot (Schwarze 2007). An extensive explanation of the decomposition patterns together with detailed drawings and high-resolution microscopic images can be found in Schwarze (2007). A comparison of the genomes of 33 basidiomycetes showed considerable differences in the number of genes encoding lignin-attacking peroxidases as well as CAZymes which target crystalline cellulose between white rot and brown rot fungi (Riley et al. 2014). In the case of peroxidases, 5 to 25 different genes were identified for white rot fungi while none were found for brown rot fungi. However, for other lignin-degrading enzymes an overlap by white and brown rot fungi was found suggesting that the current categorization needs to be more nuanced to better capture the diversity of wood decay mechanisms (Riley et al. 2014).

While the above three categories cause the main damage on wood material, there are also other fungal groups capable of colonizing wood material. Mould fungi, for examples, can also cause economic damage to wood. They colonize the parenchyma cells near the surface, but they only feed on the easily accessible nutrients, like monosaccharides and polysaccharides, proteins or fatty acids (Daniel 2016). They actually do not attack and degrade the lignolytic building blocks of wood and therefore they do not cause a loss of strength. However, they can cause permanent discolouration and thus reduce the value of the wood as construction material (Schmidt 2006).

Blue stain fungi are also frequently found on wood. They mostly belong to the ascomycetes or deuteromycetes, grow in the sapwood and cause a blue discolouration of the wood due to their brown melanin-containing hyphae (Zink and Fengel 1989). Generally, they only break down the plant cell wall in the area of the transpressorium (specialized hyphae to penetrate lignified cell walls by means of mechanical pressure and/or enzyme activity), which can lead to an increased permeability of wood without affecting its structural integrity (Schmidt 2006). Similar to mould fungi, the loss in value is mostly based on the discolouration (Schmidt 2006). However, some blue stain fungi (e.g. *Lasiodiplodia theobromae*) are also capable of producing soft rot, especially in hardwoods with tension wood (Encinas and Daniel 1995, 1997).

The diversity of wood decay fungi has already been investigated in many countries and geographical regions. Table 1 shows an overview. Huckfeldt and Schmidt (2015) associated 117 different fungal species (45 brown rot and 72 white rot) from 5140 cases of wood damage in Germany with *Serpula lacrymans* (20.8%), *Coniophora puteana* (12.8%), *Antrodia* spp. (8.6%) and *Donkioporia expansa* (7.6%) being the most common species. In addition, 12.5% were caused by soft rot fungi, which were not broken down to species level. Similar results were reported for Belgium from 407 findings resulting in 106 different identifications (83 species, 22 genera, 1 group), *S. lacrymans* (41.9%), *Donkioporia expansa* (15.8%) and *Coniophora* spp. (8.8%) were the most common once (Fraiture 2008). The majority of the investigations focused on wood decay fungi causing indoor damage and only few were done on exterior timber (Table 1). Gabriel and Švec (2017) showed that *S. lacrymans* and *C. puteana* were the most abundant indoor basidiomycetes in Europe except Norway, where the genus *Antrodia*/*Fibroporia* was dominating. *Antrodia/Fibroporia* species play an important role on exterior structures like preservative-treated utility poles due to their copper tolerance (Bollmus et al. 2012; Collett 1992; Leithoff et al. 1995; Råberg and Daniel 2009; Ribera et al. 2017). Although copper-based wood preservatives provide effective protection against most wood-decaying fungi, there are species besides *Antrodia*/*Fibroporia* such as *Rhodonia placenta* (previously *Postia placenta*), *Wolfiporia cocos*, *Pilatoporus palustris* (previously *Tyromyces palustris*) or *Meruliporia incrassata* that are able to extensively degrade copper-treated wood (Green and Clausen 2003, 2005). These types of fungi react to copper-treated wood with an increased production of oxalate (Clausen and Green 2003). The oxalate has two important effects. Firstly, it lowers the ambient pH value, which reduces the toxicity of the wood preservatives (Humar et al. 2001). Secondly, it forms crystalline, insoluble copper oxalate which is non-toxic to fungi (Humar et al. 2002). However, the formation of copper oxalate can be blocked temporarily by adding ethanolamine to the treatment solution (Humar et al. 2002). Further important fungi on exterior timber belong to the genera *Gloeophyllum* and *Dacrymyces* (Huckfeldt and Brischke 2024; Schrader et al. 2024).

**Table 1:** Overview of diversity studies focusing on wood decaying fungi affecting timber constructions

Country	Subject of investigation	Number of samples	Number of species / groups	Method of identification	Most abundant species
Austria (Haas et al. 2019)	Buildings (monumental and residential)	645	40 species and 74 genera	Macro- and microscopic, Sanger Sequencing	*Serpula lacrymans* (61.2%), *Antrodia* spp. (10.8%), *Gloeophyllum* spp. (8.2%)
Belgium (Fraiture 2008)	Buildings	407	83 species, 22 genera, 1 group	Macro- and microscopic	*S. lacrymans* (41.9%), *Donkioporia expansa* (15.8%), *Coniophora puteana* (8.8%)
Denmark (Koch 1985)	Buildings	1400	4 species, 2 genera, 2 families, 3 groups	Macro- and microscopic	*S. lacrymans* (22.5%), *C. puteana* (20.5%), *Corticiaceae* spp. (16.5%)
Estonia (Pilt et al. 2009)	Buildings	633	14 species and 2 families	Macro- and microscopic	*S. lacrymans* (79.3%), *C. puteana* (7.0%), *Antrodia* spp. (5.3%)
Finland (Paajanen and Viitanen 1989)	Buildings	1039	8 species, 1 genus	Not reported, most likely macro- and microscopic	*S. lacrymans* (53.3%), *C. puteana* (13.9%), *Antrodia sinuosa* (13.3%)
France (Maurice et al. 2011)	Buildings	74	11 species	Capillary electrophoresis single-strand conformation polymorphism (CE-SSCP) and denaturing high-performance liquid chromatography (DHPLC)	*S. lacrymans* (64%), *C. puteana*, *Trametes versicolor*, *D. expansa*
Germany (Huckfeldt and Schmidt 2015)	Buildings	5140	117 species, 11 genera, 1 family, 1 group	Macro- and microscopic, Sanger Sequencing	*S. lacrymans* (20.8%), *C. puteana* (12.8%), *Antrodia* spp. (8.6%)
Germany (Bollmus et al. 2012)	Utility poles	18	1 specimen, 1 genus	Macro- and microscopic, Sanger Sequencing	*Fibroporia vaillantii (*Syn. *Antrodia vaillantii)*, *Antrodia* spp.
Germany (Huckfeldt and Brischke 2024)	Playground structures	506	25 groups	Macro- and microscopic	*Gloeophyllum* spp. (14.4%), Corticiaceae (14.2%), *Dacrymyces* spp. (11.9%)
Germany (Schrader et al. 2024)	Playgrounds, Bridges and other exterior structures	46	129 Amplicon sequence variants (ASV)	Macro- and microscopic, Next Generation Sequencing	*Dacrymyces stillatus, Dacrymyces capitatus, Perenniporia meridionalis*
Japan (Horisawa et al. 2017)	Wooden houses	16	105 ASV	Next Generation Sequencing	*A. sinuosa, Trametes hirsuta, Hyphodontia* sp.
Latvia (Irbe and Andersone 2008)	Buildings and cultural monuments (interior and exterior structures)	338	60 species, 1 family, 1 group	Macro- and microscopic	*S. lacrymans* (46.7%), *Antrodia* spp. (*12.7%*), *Coniophora* spp. (5.9%)
Latvia (Irbe et al. 2012)	Cultural monuments (exterior structures)	90	58 species, 6 genera, 1 group	Macro- and microscopic	*Antrodia* spp., *Gloeophyllum* spp., *Athelia* spp.
Macedonia (Irbe et al. 2008)	Cultural monuments (interior structures)	44	32 species, 2 groups	Macro- and microscopic	*Hyphodontia crustosa* (11.4%), *Cladosporium* sp. (6.8%), *Hyphodontia aspera* (4.6%)
New Zealand (Stahlhut 2008)	Buildings	421	68 identifications (13 species, 7 genera, 5 groups)	Macro- and microscopic, Sanger Sequencing	*Gloeophyllum sepiarium* (19.1%), *Rhodonia placenta* (Syn. *Oligoporus placenta,* 16.2%), *A. sinuosa* (11.8%)
Norway (Alfredsen et al. 2005)	Buildings (interior and exterior structures)	3434	27 species, 3 genera, 1 family, 3 groups	Not reported, most likely macro- and microscopic	*Antrodia* spp. (18.4%), *C. puteana* (16.3%), *S. lacrymans* (16.0%)
Poland (Wazny and Czajnik 1963)	Buildings	3050	29 species	Macro- and microscopic	*S. lacrymans* (52.3%), *C. puteana* (22.4%), *Antrodia* spp. (12.4%)
South Korea (Kim et al. 2005)	Playground	Not reported (35 playgrounds)	132 isolations comprising 32 species	Microscopic and Sanger Sequencing	*Schizophyllum commune* (15.2%), *T.s versicolor* (15.2%), *G. trabeum* (9.1%)
Sweden (Råberg and Daniel 2009)	Fence posts	Not reported	2 species, 1 genus, 1 group	Microscopic, Sanger Sequencing and inter-compatibility tests	*F. vaillantii (*Syn. *Antrodia vaillantii)*
Switzerland and Germany (Ribera et al. 2017)	Utility poles	111	8 species, 1 group	Macro- and microscopic	*Neoantrodia serialis* (Syn. *Antrodia serialis) F. vaillantii*, *Serpula himantoides*
USA (Wilcox and Dietz 1997)	Buildings (interior and exterior structures)	103	12 species, 1 group	Microscopic	*Antrodia carbonica* (22.3%), *G. trabeum* (15.5%), *G. sepiarium* (14.6%)

### Bacteria

Besides fungi, bacteria also have the ability to degrade wood. While fungi play a dominant role in the decomposition of wood above ground or in soil contact, bacterial decomposition becomes more relevant under environmental conditions, where fungal activity (except soft rot fungi) is suppressed due to limited oxygen availability, such as buried wood or submerged under water (e.g. Björdal et al. 1999; Holt and Jones 1983; Kim et al. 1996; Schmidt and Liese 1994). But there is also another adverse impact of bacteria on timber wood, which relates to the capacity to degradation wood preservatives (e.g. Daniel and Nilsson 1985; Greaves 1968; Singh and Wakeling 1997). The resulting detoxification of preservatives can then facilitate a subsequent fungal wood decay (Mai et al. 2004; Schmidt and Liese 1994; Wallace and Dickinson 2006). Although a bacteriostatic effect of the preservatives has been proven in the laboratory, the failure of treated timber in use shows the limitations of such experiments (Greaves 1973; Liese and Schmidt 1975; Edlund and Nilsson 1999). In the case of metal-containing preservatives, it is hypothesized that the heavy metals could be inactivated by extracellular bacterial slime secretion and formation of a non-toxic complex (Daniel et al. 1987; Greaves 1971). Schmidt and Liese (1994) stated that the reduced efficacy under environmental conditions was caused by acids produced by bacteria which led to a lower pH value, so that fixed preservatives were lost from the wood by leaching. Clausen (2000b) was able to isolate 13 different metal-tolerant bacterial species, including *Acinetobacter calcoaceticus*, *Aureobacterium esteroaromaticum*, *Klebsiella oxytoca* and *Bacillus licheniformis*. In the case of organic wood preservatives, detoxification was often associated with the Gram-negative *Proteobacteria*, especially from the genus *Pseudomonas* as well as *Alcaligenes*, *Enterobacter* but also with *Microbacterium*, a member of the phylum Actinobacteria (Cook et al. 2002; Wallace et al. 2008).

Two forms of bacterial wood decomposition are described: tunnelling and erosion (reviewed in Blanchette et al. 1990; Kim and Singh 2000; Singh and Butcher 1991). Both forms are solely based on the microscopically tangible decay patterns of the wooden cell wall and both are not directly linked to specific taxonomic groups (Daniel 2014; Singh et al. 2016). Sometimes cavitation bacteria are distinguished as a third decomposition form (Clausen 1996; Kim and Singh 2000). Tunnelling bacteria can penetrate the plant cell wall from the cell lumen or the wood surface and produce branching, convoluting tunnels as shown by transmission electron microscopy (Daniel 2014; Daniel and Nilsson 1985). Erosion bacteria degrade the plant cell wall starting from the lumen by producing troughs that are parallel to cellulose microfibrils (Kim and Singh 2000). In contrast to tunnelling bacteria, they do not degrade the lignified middle lamella, which shows their limiting ability to degrade lignin (Daniel 2014). Cavitation bacteria produce diamond-shaped cavities which are perpendicular oriented to the long direction of the fibre and start to form near pit chambers or directly within the second layer of the plant cell wall (Singh and Butcher 1991).

## Methods of identification

Early detection and identification of wood decay organisms are of crucial importance for preventing major damage as well as choosing proper sanitation methods. In Germany, before a fungal infestation can be remediated, the causing fungus must be determined to such an extent that an infestation by *S. lacrymans* can be ruled out (Deutsches Institut für Normung 2020). Wood-decaying fungi can be identified based on morphological features of their fruit bodies (e.g. Gminder et al. 2000, 2001, 2003; Gminder and Kriegelsteiner 2010; Huckfeldt and Schmidt 2015; Kriegelsteiner and Kaiser 2000). However, fruitbodies are often missing or in bad condition; in that case, some fungi can be identified based on their formation of mycelial strands (Falck 1912; Huckfeldt and Schmidt 2006, 2015). Other identification keys are based on the mycelium grown on agar, which requires successful isolation and cultivation of the decay agent (Nobles 1965; Stalpers 1978). Additionally, for some genera, i.e. *Antrodia*, *Coniophora* or *Lentinus*, species differentiation solely based on strands and visible mycelia is not possible (Huckfeldt and Schmidt 2015). Furthermore, morphological identification requires a lot of experience; otherwise, misidentification can happen (Horisawa et al. 2004; Schmidt et al. 2002).

In many cases of damage, neither fruiting bodies nor mycelia are present. Therefore, a variety of molecular methods have been invented to identify mostly indoor basidiomycetes. Some methods were protein based like polyacrylamide gel electrophoresis (Schmidt and Kebernik 1989; Schmidt and Moreth 1995) and matrix-assisted laser desorption/ionization time-of-flight mass spectrometry (Horisawa and Iwamoto 2022) or enzyme-linked immunosorbent assays (Clausen et al. 1991; Jellison and Goodell 1988).

DNA-based methods utilizing PCR-techniques offer a great potential for the detection and identification of wood-degrading microorganisms. Numerous DNA-based techniques were already developed for the identification of indoor basidiomycetes. These include taxon-specific priming PCR (Guglielmo et al. 2008; Horisawa et al. 2009; Moreth and Schmidt 2000), random amplified polymorphic DNA (RAPD) analysis (Hseu et al. 1996; Schmidt and Moreth 1998), restriction fragment length polymorphism (RFLP) analysis (Jasalavich et al. 2000; Schmidt and Moreth 1999), DNA microarray technology (Jacobs et al. 2010), low-cost and low-density (LCD)-macroarray technology (Ragno et al. 2017), melting curve analysis (Horisawa et al. 2013) and direct sequencing of the internal transcribed spacer region of ribosomal DNA (Högberg and Land 2004; Moreth and Schmidt 2005; Schmidt and Moreth 2002).

All of the abovementioned DNA-based methods require a pure sample of a single microorganism for DNA extraction. This, however, can be challenging to get because environmental samples are often colonized by various coexisting microorganisms. One way to enhance the detection of wood-decaying fungi can be the addition of growth retardants or inhibitors like thiabendazole, benomyl or streptomycin to the agar medium which prevents overgrowth of co-occuring bacteria, mould or soft rot fungi (Ribera et al. 2017; Råberg and Daniel 2009). An overview of different growth retardants can be found in Crous et al. (2009).

Moreover, all of the previous methods only enable the identification of a restricted number of species mostly the more common indoor wood basidiomycetes (Schmidt 2007). To overcome some of the restrictions of the named DNA-based methods, Maurice et al. (2011) used capillary electrophoresis single-strand conformation polymorphism (CE-SSCP) to identify *S. lacrymans* and 16 other wood-decaying basidiomycetes from environmental samples. By using denaturing high-performance liquid chromatography (DHPLC) with subsequent sequencing of the amplicons, they were also able to analyze parts of the fungal community infecting environmental samples (Maurice et al. 2011).

Since the 2000s, the rapid improvement of next-generation high-throughput sequencing technologies facilitated the analysis of DNA from various environmental samples, such as air, soil, water, faeces and wood (Ansorge 2009; Shendure and Ji 2008; Taberlet et al. 2012b), which made it possible to characterize entire microbial communities, identify important biochemical functions via the analysis of coding genes or assemble whole genomes of yet uncultured microorganisms (Ellwood et al. 2010; Taberlet et al. 2012a; Tedersoo et al. 2014; Tláskal et al. 2021). Although next-generation platforms increased the sequencing capacity, they are only able to process shorter amplicons (< 550 bases) compared to Sanger sequencing (~ 700 bases, Kauserud 2023; Tedersoo et al. 2022).

The most important genetic marker to identify fungi based on their phylogeny is the genomic ITS region. Until recently, current PCR-based sequencing protocols could not obtain the full ITS sequence, which typically has a length of 450 to 750 nucleotides (Blaalid et al. 2013). It was therefore necessary to amplify only sub-regions of the ITS, i.e. the ITS1 or ITS2, which ultimately resulted in lower taxonomic resolution and loss of phylogenetic information, including some genera also relevant for identifying wood-decaying fungi (Badotti et al. 2017; Tedersoo et al. 2022). The advent of the so-called “third generation high-throughput sequencing platforms” today provides the new opportunity to massively sequence longer amplicons with great accuracy. This allows to retrieve information on the entire ITS region and thereby increases the ability to identify fungi (Wurzbacher et al. 2019). However, it is challenging to obtain longer amplicons with high quality from some environmental samples (Kauserud 2023; Tedersoo et al. 2021). Some studies have successfully applied third-generation sequencing to analyze the fungal communities of deadwood targeting the full ITS region (Purahong et al. 2019, 2024). It should be noted that the various techniques reported are all based on directly extracted DNA from environmental samples and also on PCR amplifications of target gene sequences and these approaches can be biased, making it sometimes difficult to compare results from different studies. For instance, there can be a bias caused by different DNA extraction methods, a bias due to different cell lysis protocols, PCR bias caused by the use of different primers or the same primers binding with different specificities, the generation of chimera PCR amplicon sequences (combined sequence of two different fungi), or amplicon index switching in the context of preparing the samples for sequencing. All of these potential sources of errors for the cultivation-independent detection of microbial communities have been reviewed extensively (e.g. Kauserud 2023; Nilsson et al. 2019; Tedersoo et al. 2022).

It must also be emphasized that regardless of the DNA sequencing platform used, the identification fully depends on the presence of sequences in the reference databases, i.e. UNITE, Warcup or INSD (Abarenkov et al. 2024; Deshpande et al. 2016). A successful identification is based on the presence of sequences of correctly identified species in the databases. In other words, sequences of species that were misidentified or not present might be incorrectly assigned (Nilsson et al. 2006; Schrader et al. 2024). Estimations of global fungal diversity range from 700,000 (Schmit and Mueller 2007), over 2.2–3.8 M (Hawksworth and Lücking 2018), 5.1 M (Blackwell 2011) to 6.2 M species (Baldrian et al. 2022) while for example the UNITE database currently contains almost 4 Million sequences representing ca. 200,000 fungal species which shows that the coverage of reference databases is still limited and species annotation must be critically evaluated. While the DNA-based approaches described here provide overall a fundamental, indispensable level of information, the results should not be blindly trusted, due to the limitations mentioned above, and thus, these molecular identification methods should be combined with traditional morphological methods whenever possible for a solid and unequivocal identification.

The impact of DNA-based, culture-independent, technologies on bacterial ecology research was even greater as compared to fungi because in contrast to fungi, the proportion of non-yet cultured or culturable bacteria is much higher (Handelsman 2004; Tringe and Rubin 2005). As of today, only few studies have been carried out targeting wood-degrading bacterial communities, and the majority of those focussed on litter or deadwood and not on timber (reviewed in Johnston et al. 2016). One of such studies reported that in early deadwood stages, bacterial communities were dominated by taxa with an increased potential to utilize cellulose, i.e. members of the phyla *Acidobacteria, Bacteroidetes* and *Actinobacteria* (Tláskal and Baldrian 2021). In later decay stages, they are replaced by opportunistic bacteria, mostly belonging to *Alphaproteobacteria* and *Gammaproteobacteria*, and these bacteria are suspected to rely on by-products from fungal degradation or mycophagy. This detection of community successions in fact correlates well with findings from bacterial communities on degraded timber (Schrader et al. 2024; Tláskal and Baldrian 2021).

While attempts to grow pure cultures from bacteria inhabiting waterlogged wood have failed (Nilsson and Björdal 2008; Nilsson and Daniel 1992), consortia of mixed bacteria, based on PCR amplicon analyses from directly extracted DNA could be obtained and used to reproduce tunnelling and erosion attack on sound wood (Nilsson and Daniel 1992). The analysis of purified cultures as well as environmental samples of waterlogged wood with DNA-based methods found that the majority of bacteria were members of the *Cytophaga*-*Flavobacterium*-*Bacteroides*-complex, as well as *Pseudomonas, Cellvibrio* and *Brevundimonas* (Landy et al. 2008; Nilsson et al. 2008).

Overall, the DNA-based analyses already demonstrated their great potential for enhancing our understanding of the microbial, and especially the bacterial attack on timber wood. Yet this information is still too sporadic to identify and predict the wood-microbial interactions at larger scales, the impact of wood material and environmental conditions as defined by their use, and also by the biogeographical region in which it is used.

## Wood protection

Wood protection covers all aspects that are intended to extend the service life of wood products and deals with protection against fire, chemical degradation, weathering, mechanical wear and biological attack (van Acker et al. 2023) mainly caused by insects, marine borers, fungi and bacteria. Here, we focus on methods and technologies to protect wood against the degradation by microorganisms, including both fungi and bacteria. The majority of such methods are, in fact, directed against fungi due to their outstanding role in limiting the sustainable use of timber wood.

### Wood decay influencing factors

Wood decomposition by fungi is mainly influenced by wood moisture content, temperature and oxygen availability (Schmidt 2006). The limitation of oxygen to control fungal colonization and growth is no option, neither for indoor nor for outdoor constructions (Cappellazzi et al. 2020). However, it can be used to prevent fungal colonization during storage in specifically controlled rooms (Metzler et al. 1993) or to eradicate infestations of valuable wooden objects like sculptures or works of art without destroying them (Tavzes et al. 2003).

In the case of temperature, the minimum requirement for fungal growth is just above 0 °C (Huckfeldt et al. 2005), because liquid water is needed for the enzymatic reactions (Schmidt 2006). In the case of wood degradation, Wälchli (1977) measured 1.8% mass loss after 16-week incubation at 3 °C caused by *C. puteana* which increased to 17.9% at 8 °C. The optimal temperatures ranged between 22 and 33 °C depending on the fungal species (Wälchli 1977). While the temperature for outdoor structures cannot be influenced at all, indoors, it usually lies within a suitable range for wood degradation.

Wood moisture content is the factor that can actually be influenced best to prevent fungal degradation. In general, fungal mycelium is not able to absorb water which is bound within the plant cell wall due to osmotic pressure (Schmidt 2006). For the transport of released enzymes from the hyphae to the plant cell wall as well as breakdown products back to the hyphae free water is needed, so the minimum moisture content needs to be above cell wall saturation which is at around 25% (Brischke & Alfredsen 2020; Schmidt 2006; Zabel and Morrell 2020). Some basidiomycetes are able to colonize wood well below cell wall saturation, because they can transport water using their mycelium if a moisture source is near (Huckfeldt and Schmidt 2015; Meyer and Brischke 2015; Stienen et al. 2014). The equilibrium moisture content of wood used indoors ranges between 6 and 19% which is too low for fungal decay and therefore not at risk (Cappellazzi et al. 2020). For wood exposed outdoors, critical moisture contents above 25% are reached, but the number of days above the threshold (time of wetness, ToW) depends on the wood species, location and the design details (Brischke and Meyer-Veltrup 2015; Brischke and Rapp 2008; Isaksson and Thelandersson 2013).

### Wood protection by design

Wood used indoors is only susceptible to fungal decay if water traps are formed, e.g. by leaky insulation, leakages in bathrooms or damage of the building envelope during installation (Brischke et al. 2006). Wood used outdoors is exposed to a significantly higher risk of infestation, which makes the proper design even more important. The aim is to keep the wooden parts at a low moisture content, e.g. by avoiding direct soil contact as a source of moisture, improved water drainage through bevelling or declination, roofing with sufficient overhang or covering load-bearing components (Brischke et al. 2006; Huckfeldt 2011). Where wetting is inevitable, wood of high natural durability, modified or preservative-treated wood should be used (Cappellazzi et al. 2020). The durability against wood-decaying organisms can be determined in laboratory resistance tests using pure cultures of wood decay fungi or in field tests. Marais et al. (2022) provide an overview of testing methods used in different continents.

### Wood preservation

Wood can be treated with chemical biocides to protect it against biodegradation by fungi, bacteria, insects or marine borers and, thus, extend its service life. Biocides can also reduce costs by decreasing the efforts for maintenance, repair or replacements of wooden parts (Khademibami and Bobadilha 2022; Kirker and Lebow 2021). Generally, chemical wood preservatives can be distinguished in two classes, i.e. oil-borne and water-borne preservatives (Kirker and Lebow 2021).

Commonly and widely applied oil-borne preservatives like creosote and pentachlorophenol (PCP) have historically been used for treating railway sleepers, bridge timber or utility poles (Brient et al. 2020). Due to their negative environmental impact, the use of creosote is mostly restricted to treating railway sleepers and utility poles while PCP was added to the Annex A of the list of persistent organic pollutants of the Stockholm convention and can only be used for treating utility poles and cross-arms (Jurys et al. 2015; Sved et al. 1997; United Nations 2015b; Wang et al. 2001; Wegner et al. 2024). In North America, oil-borne preservatives with copper have been used as an alternative for many years, while in Europe, this has only recently begun (Brient et al. 2020; Wegner et al. 2024). Since some fungi are copper-tolerant, co-biocides are often required to achieve a more comprehensive protection (Freeman and McIntyre 2008; Schmidt and Moreth 1996).

Water-borne preservatives mainly contain copper as a biocide, but a co-biocide must also be used to avoid attack by copper-tolerant fungi (Kirker and Lebow 2021). The water-borne preservative that was used the most since the 1970s was chromated copper arsenate (CCA, Clausen 2000a; Freeman et al. 2003). In Europe, the use of CCA is now banned, as arsenic has an extremely negative impact on the environment through leaching into the soil or water (Katz and Salem 2005; Kirker and Lebow 2021; Morais et al. 2021). Alternatively, other copper-based preservatives like copper azole, alkaline copper quaternary or copper naphthenate are used (Schultz et al. 2007; Khademibami and Bobadilha 2022). Nowadays, micronized copper preservatives play an important role. Copper is not dissolved in the solution as usual, but nanoparticles with the size between 10 and 700 nm (median particle size 100–200 nm) of copper carbonate are added to the treatment solution (Johnson et al. 2021; Zelinka et al. 2022b). In 2009, typical micronized copper systems like micronized copper azole and micronized copper quaternary constituted approximately 80% of the pressure-treated lumber sold for residential construction in the USA (Cushman 2009).

In addition to copper, inorganic boron is used as a biocide in water-borne wood preservatives (Kirker and Lebow 2021). Several formulations containing borates have been shown to be effective against wood decay fungi (Lyon et al. 2009; Mohamad-Nasir et al. 2019; Thévenon et al. 1997). However, borates are easily leachable which is why treated products are mainly recommended for indoor use or protected, while at the same time, new fixation methods are investigated (Ibañez et al. 2021; Mohareb et al. 2011; Thévenon et al. 2009). However, the use of boron compounds in the EU is restricted, as they have been classified as substances of very high concern in 2010 (Wegner et al. 2024).

### Wood modification

Wood modification encompasses the application of chemical, physical or biological processes to alter the properties of wood (Hill 2006). The goal of the majority of wood modification methods is to enhance the decay resistance against decay fungi and insects. Simultaneously, the modification can improve the dimensional stability, decrease the hydrophilicity of wood and impact mechanical properties (Zelinka et al. 2022a). The commercially most important forms of modification are thermal modification, acetylation, furfurylation and resin impregnation/polymerization (Jones and Sandberg 2020).

Thermal modification of wood refers to processes that involve heating wood at temperatures between 160 and 240 °C in an oxygen-controlled environment (Hill et al. 2021; Jones and Sandberg 2020). Since the 1990s, several processes have been developed and used for the commercial production of thermally modified timber (TMT); of that, more than 500,000 m^3^ are now produced annually in Europe (Hill et al. 2021; Jones et al. 2019). The different processes differ in treatment atmosphere (i.e. partial vacuum, steam atmosphere, hot oil, inert gas atmosphere), treatment temperature and duration (Gérardin 2016; Zelinka et al. 2022a). The heat treatment alters the internal chemical composition (i.e. degradation of hemicelluloses and amorphic celluloses, depolymerization and recondensation of lignin), which results in several positive properties such as increased resistance to fungal decomposition in above-ground situations, improved dimensional stability and reduced moisture uptake (Altgen and Militz 2016; Burmester 1975; Sivonen et al. 2002; Tjeerdsma et al. 1998; Welzbacher et al. 2007). However, the chemical changes have a negative effect on the strength properties of the modified wood (Boonstra et al. 2007; Kubojima et al. 2000).

For acetylation, wood is treated with acetic anhydride (Hill 2006). The reaction with acetic anhydride results in esterification of the accessible hydroxyl groups in the cell wall with the formation of acetic acid as a by-product (Rowell et al. 1994). This is a single-site reaction, which means that one acetyl group is replacing one hydroxyl group with no polymerization happening (Rowell 2007). This change has two consequences: firstly, a bulking effect of the cell wall, as the acetyl group is larger than the hydroxyl group, and secondly, a lower hydrophilicity as the acetyl group has a low polarity (Zelinka et al. 2022a). This results in a decreased moisture uptake and increases the resistance to swelling as well as decay by fungi (Bollmus et al. 2015; Larsson-Brelid and Westin 2007; Larsson-Brelid et al. 2000; Jones and Sandberg 2020). However, the mechanisms behind the protection of acetylated wood are not yet fully understood and have been discussed in several reviews (Ringman et al. 2019, 2014; Zelinka et al. 2016, 2022a).

Furfurylation is a process where wood is impregnated with furfuryl alcohol and maleic anhydride, citric acid or other weak acids as catalysts, followed by a heat-curing step to enable polymerization of the furfuryl alcohol (Jones and Sandberg 2020; Nordstierna et al. 2008; Schneider 1995; Westin et al. 1996; Zelinka et al. 2022a). Inside the plant cell lumen, a furan polymer is formed which binds to lignin but not to cellulose or hemicellulose (Barsberg and Thygesen 2017; Lande et al. 2008; Nordstierna et al. 2008; Shen et al. 2021; Thygesen et al. 2010). At high modification levels, a variety of wood properties is enhanced by furfurylation, i.e. increased hardness, modulus of rupture, modulus of elasticity, dimensional stability and resistance to microbial decay (Lande et al. 2004; Vetter et al. 2009).

Further modification methods to increase the resistance of wood against microbial decay use thermosetting resins like phenol formaldehyde and melamine formaldehyde or cyclic N-methylol compounds such as 1,3-dimethylol-4,5-dihydroxyethyleneurea (Behr 2020; Biziks et al. 2021; Emmerich et al. 2019, 2021; Krause 2006). The modification with high chemical loadings of sorbitol and citric acid also generated adequate protection against wood decay fungi (Belt et al. 2023; Kurkowiak et al. 2023). In addition, modification with organic and inorganic silicon compounds offers a further way of protecting wood from degradation by fungi and insects (Emmerich et al. 2022; Weigenand et al. 2008).

In terms of global production, thermal modification is the most important method producing 1,110,000 m^3^/year (Europe, 695,000 m^3^; China, 250,000 m^3^; North America, 140,000 m^3^; Oceania / Japan, 15,000 m^3^; Other, 10,000 m^3^), followed by acetylation with 120,000 m^3^/year (only Europe) and furfurylation with 45,000 m^3^/year (only Europe), while other processes combined account for 330,000 m^3^/year (Europe, 35,000 m^3^; China, 290,000 m^3^; Oceania / Japan, 5000 m^3^; Jones and Sandberg 2020). However, compared to the amount of preservative-treated wood in the USA (21 million m^3^/year) or Europe (6.5 million m^3^/year), modified wood is despite its growing production still a niche product (Zelinka et al. 2022a).

For in-depth information on the subject of wood modification, numerous review papers are available (Gérardin 2016; Hill et al. 2021; Jones and Sandberg 2020; Kurkowiak et al. 2022; Zelinka et al. 2022a).

## Outlook

As climate change progresses, the use of wood as a building material is becoming an important means of reducing emissions in the construction sector. It is equally important not only to use more wood but also to extend its useful life in order to maximize resource conservation and sustainability. Accordingly, comprehensive knowledge about the distribution and importance of wood-degrading organisms is desirable. Monitoring programmes could provide knowledge about the frequency and distribution of different species and thus about the expected risk regarding the functionality and service life of wooden components. Decay types and, in some cases, individual fungal species have different effects on the structure of the wood and its strength properties. Fungal monitoring could therefore help to identify problematic species that more often cause damage. Based on the knowledge of the ecology of these fungal species, it would be possible to develop proposals for improving wood protection by design. In addition, newly emerging pests could be identified, whether due to climate change or global trade. In addition to fungi, this also applies to other threats such as termites or marine borers. Other ways to increase the service life of wood are wood preservation and wood modification. As this review shows, there are already numerous possibilities for producing environmentally friendly, durable wood or wood materials.
